# Successful transcatheter stabilization for both acute mitral regurgitation and ruptured papillary muscle following acute myocardial infarction

**DOI:** 10.1093/ehjcr/ytae225

**Published:** 2024-04-29

**Authors:** Masanori Yamamoto, Ai Kagase, Takahiro Tokuda

**Affiliations:** Department of Cardiology, Nagoya Heart Center, 1-1-4 Sunadabashi, Higashi-ku, Nagoya, 461-0045 Aichi, Japan; Department of Cardiology, Toyohashi Heart Center, 21-1 Gobutori, Oyamachyo, Toyohashi, 441-8530 Aichi, Japan; Department of Cardiology, Gifu Heart Center, 4-14-4 Yabutaminami, Gifu city, 500-8384 Gifu, Japan; Department of Cardiology, Nagoya Heart Center, 1-1-4 Sunadabashi, Higashi-ku, Nagoya, 461-0045 Aichi, Japan; Department of Cardiology, Nagoya Heart Center, 1-1-4 Sunadabashi, Higashi-ku, Nagoya, 461-0045 Aichi, Japan

A 92-year-old female was emergently transferred to our hospital due to sudden onset mitral regurgitation (MR) following acute myocardial infarction (AMI) with cardiogenic shock. The AMI culprit lesion was located in the left circumflex artery of segment 13 and was treated by drug-eluting stent implantation percutaneously. Transoesophageal echocardiography (TEE) revealed severe MR with a ruptured papillary muscle (PM) and posterior mitral leaflet prolapse of the P3 segment (*Figure 1A* and *B*). Given the patient’s advanced age and unstable condition, emergent transcatheter edge-to-edge repair (TEER) was performed using MitraClip (G4-XT device, Abbott Vascular, USA). Although the MR degree reduced less than moderate after clipping, the ruptured PM was floated in the left atrium (LA) side above the clip (*Figure 1C* and *D*). Cardiac arrest unexpectedly occurred following tachycardiac atrial fibrillation. Upon restoration of cardio-pulmonary circulation, the operator repositioned the clip; the entire clip system, including the connecting device shaft, was sifted towards the medial side of the left ventricle (LV) (*Figure 1E*). The clip was then repositioned to a more medial site, allowing it to successfully grasp both mitral valve leaflets (*Figure 1F*). Subsequently, TEER was conducted successfully, resulting in the maintenance of MR with mild (*Figure 1G*). Additionally, the ruptured PM remained inside the LV and did not appear in the LA side after the second attempt of clipping (*Figure 1H* and *I* and Supplementary material online, *Video S1*). The patient could be discharged alive and continually caring in the rehabilitation centre at 2 months after initial TEER.

**Figure 1:**
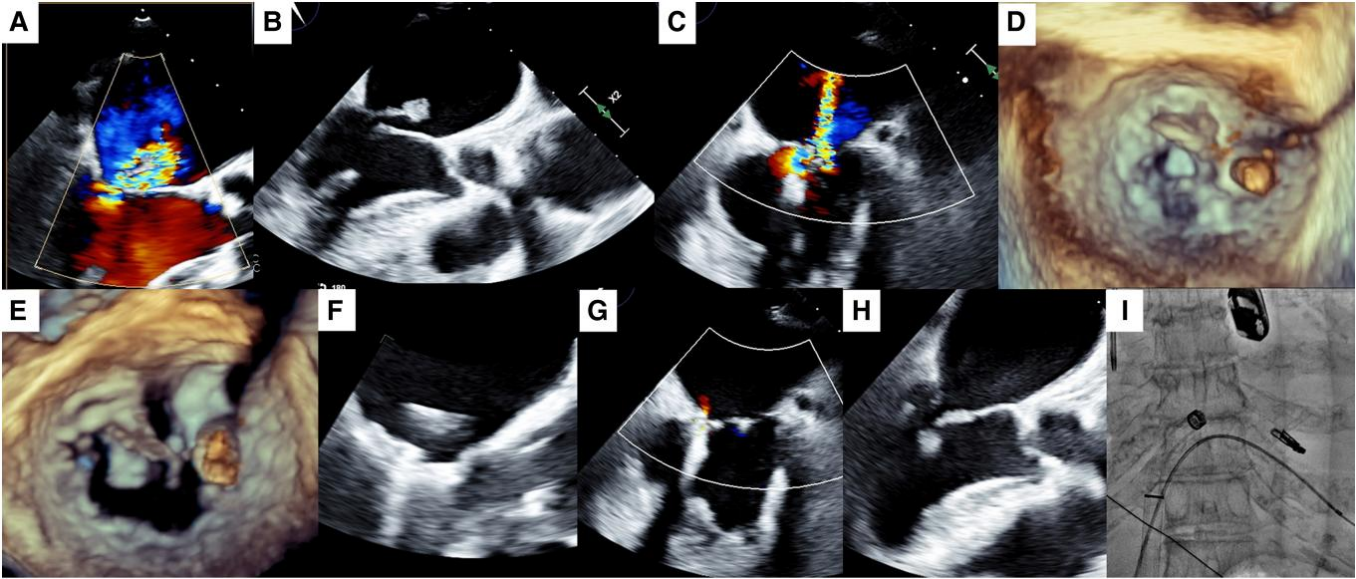
(*A*) Severe mitral regurgitation (MR); (*B*) with ruptured papillary muscle (PM); (*C*) MR after the initial attempt; (*D*) PM in the left atrium (LA); (*E*) changing the position of device; (*F*) clip attached to both the leaflets; (*G*) MR after the second attempt; (*H*) remained the PM in the left ventricle; (*I*) final angiographic image after clipping.

Although the indication of cardiac support device in patients undergoing emergent TEER was still in debate,^1^ the intra-aortic balloon pumping and temporary pacing were required during procedure. The early introduction of support device may be considered such as acute MR with PM rupture following AMI. The careful TEE observations demonstrated that stabilized acute severe MR and confined the ruptured PM within the LV without dynamic mobility. This case highlights the importance of multi-disciplinary team approach, even in emergent situations, to ensure adequate TEER execution.

## Supplementary Material

ytae225_Supplementary_Data

## Data Availability

The data underlying this article are available in the article and in its online Supplementary material.

## Supplementary material

**Consent:** Written informed consent was obtained from the patient for publication of this case report and accompanying images.
